# Developing Iranian sub-national primary health care measurement framework: a mixed-method study

**DOI:** 10.1186/s13690-023-01108-0

**Published:** 2023-06-01

**Authors:** Ramin Rezapour, Ardeshir Khosravi, Mostafa Farahbakhsh, Elham Ahmadnezhad, Saber Azami-Aghdash, Jafar Sadegh Tabrizi

**Affiliations:** 1 Tabriz Health Services Management Research Center, Tabriz University of Medical Sciences, Tabriz, Iran; 2 Deputy for Public Health, Ministry of Health and Medical Education, Tehran, Iran; 3 Department of Psychiatry, School of Medicine, Tabriz University of Medical Sciences, Tabriz, Iran; 4 National Institute of Health Research (NIHR), Tehran University of Medical Sciences (TUMS), Tehran, Iran; 5 Medical Philosophy and History Research Center, Tabriz University of Medical Sciences, Tabriz, Iran

**Keywords:** Primary Health Care, Iran, Performance measurement, Framework, Key performance indicators, Universal Health Coverage

## Abstract

**Background:**

Desired health outcomes are more achievable through strong Primary Health Care (PHC). Using comprehensive and scientific tools, decision-makers are guided to formulate better PHC reforms and policies. This study introduces a sub-national framework based on the World Health Organization (WHO) proposed frameworks for the PHC performance measurement.

**Method:**

By a mixed-method and qualitative approach, the Iranian sub-national PHC Measurement Framework (PHCMF) was developed through a review of the WHO’s PHC measurement conceptual framework (for selecting Key Performance Indicators (KPIs)), literature review (academic database), PHC-related national documents, consultations with an advisory committee of national experts (6-meetings), and the Delphi technique for finalizing the framework.

**Results:**

The Iranian sub-national PHCMF was finalized with 100 KPIs in three components including Health systems determinants, Service Delivery, and Health system objectives. Based on the result chain domain, most KPIs were related to the output (24 KPIs) and the least were related to the input and the process (9 KPIs).

**Conclusion:**

Regarding the comprehensiveness of the developed measurement framework due to its focus on all PHC operational levers and key aspects of PHC systems’ performance, it can be used as a practical tool for assessing and improving the Iranian sub-national PHC system.

**Supplementary Information:**

The online version contains supplementary material available at 10.1186/s13690-023-01108-0.

## Background

Demand for high-quality health-care services, changing population health needs, and rising health costs are the most pivotal factors that forced the health system leaders toward strengthening Primary Health Care (PHC) [1]. PHC is a critical platform for achieving an efficient, effective, and equitable health system [2]. Strong PHC has recently been emphasized as a core strategy for improving population health and achieving Universal Health Coverage (UHC), especially in Low and Middle-Income Countries (LMICs) [2–4]. In addition, on the 40th anniversary of the Almaty Declaration, the 2018 Astana Declaration, PHC was introduced as the center of the service delivery system and its fundamental and multisectoral role in improving people’s health was reconfirmed [4].

Despite a global political commitment to PHC’s central role, PHC is not a priority in many LMICs, and its performance has been poorly assessed [1, 5]. Better assessment based on strong data, information and evidence are necessary for improving and strengthening PHC [6]. Therefore, assessing the PHC performance and identifying its challenges and weaknesses is a critical step in moving towards a strong and sustainable PHC [6]. In this regard, the Primary Health Care Performance Initiative (PHCPI), a collaboration of the World Health Organization (WHO), the World Bank (WB), and the Bill and Melinda Gates Foundation, in partnership with Ariadne Labs introduced a conceptual framework in 2015 to better understand PHC performance aspects and fill the performance assessment gaps in PHC [1, 6]. PHCPI was launched to accelerate progress toward UHC through a more comprehensive and more practical quality assessment of PHC [6]. Countries have recently revised their health care systems based on the new PHC [6]. According to the Astana Declaration, the Eastern Mediterranean Regional Office (EMRO) introduced the Primary Health Care Measurement and Improvement Initiative (PHCMI) as a regional plan for implementation in region countries [7]. Using the PHCPI experience, the PHCMI has provided a set of tools and Key Performance Indicators (KPIs) for assessing the performance of PHC, based on which member countries can identify the weaknesses, strengths, and challenges of PHC (details in method section) [1, 7].

EMRO suggested that member countries assess their PHC based on KPIs [7]. Iran is one of the member countries that participated in the introduced initiative and has started the PHCMI program since 2019 and completed the first phase in 2020. Based on the initiative, the next step for Iran was to assess the sub-national level based on KPIs.

PHC in Iran is provided at three levels: national, provincial, and district [8]. PHCMI introduced KPIs for assessing PHC performance at the national level [7]. There are some differences between national level of PHC with the second and third level in Iran. So it was necessary to adjust the setting of national KPIs to the others levels. Developing a provincial and district framework to measure PHC, will provide the necessary information to compare the performance of different provinces and districts and develop improvement plans according to the specific conditions and characteristics of the sub-national levels.

The performance of the PHC system in Iran is measured using a variety of programs, but they lack the coherence and comprehensiveness to measure all PHC monitoring levers, including PHC capacity, PHC performance, and PHC impact. The aim of this research is to develop a thorough and scientific framework for measuring all PHC monitoring levers based on local and international experiences. The data generated by using this framework can allow health system management a thorough understanding of current PHC performance at the national and local levels. This study was conducted to develop a framework to measure the performance of the Iranian sub-national PHC through an adjusted framework from WHO (PHC Measurement Conceptual Framework (PHCMCF)).

## Method

### Study design

This was a mixed method study. Five steps were carried out in order to develop the Iranian Sub-national PHCMF. At first, the framework provided by WHO was reviewed and adjusted according to the Iranian PHC context at the sub-national level. Then, literature review was done to identify related PHC performance indicators. In the third step, six expert meetings were held to review related KPIs and introduce alternative KPIs. The next step was done to select the final KPIs using the Delphi technique. Then, final expert meetings were conducted to review selected KPIs and develop the Iranian Sub-national PHCM framework.

### Setting: Iranian sub-national PHC system

**Fig. 1:**
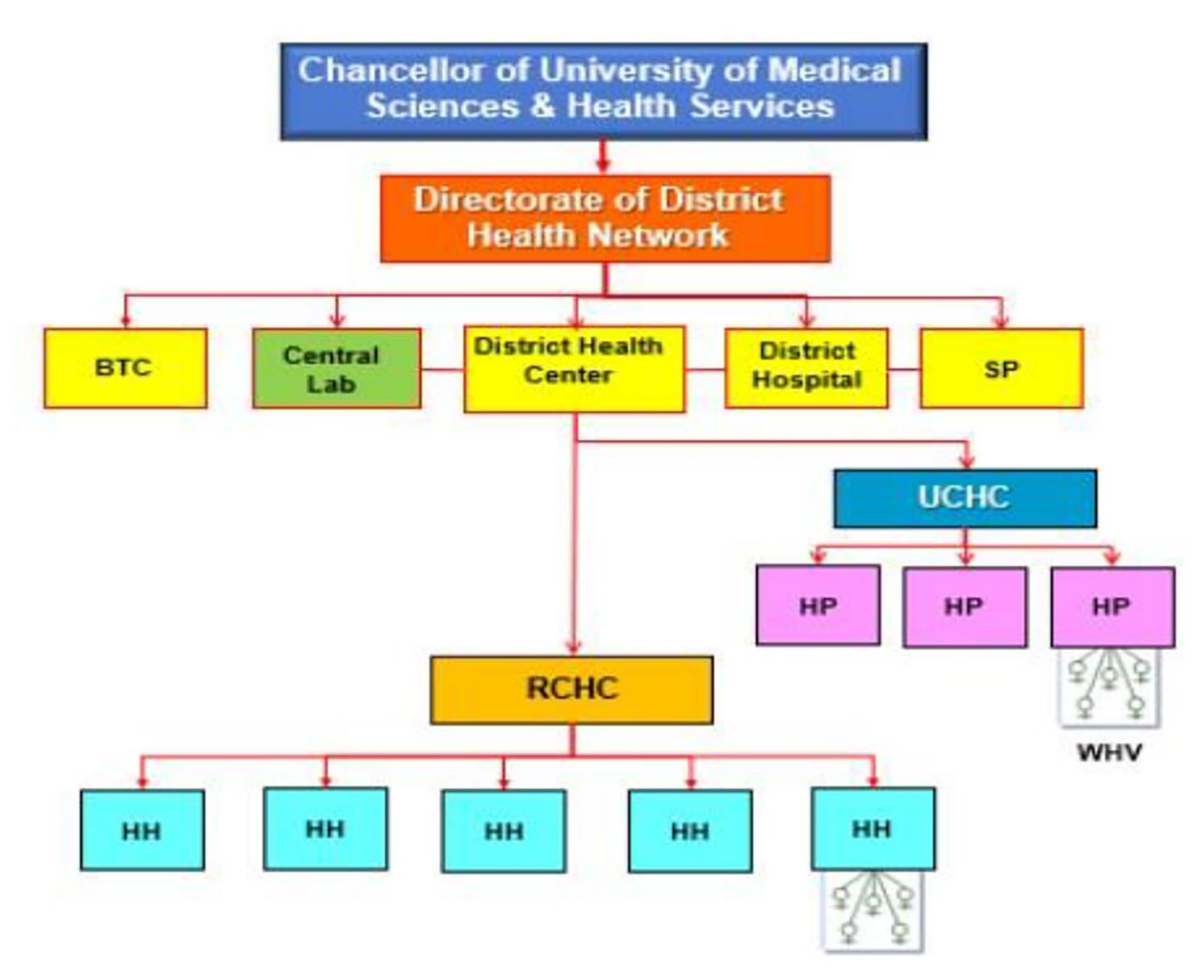
The structure of the PHC system in the Islamic Republic of Iran [9]

In Iran, there are three levels for PHC: national, provincial, and district levels. In this study, the provincial and district levels are considered sub-national levels. At the provincial and district levels, there are provincial health centers and district health networks, respectively. The Iranian PHC network was established across the country about 35 years ago. In rural areas, there is a Health House (HH) for each village or group of villages which is staffed by trained community health worker “Behvarz” [9, 10]. In addition, there are Rural Comprehensive Health Centers (RCHC) in rural areas to cover around 5 HHs. The RCHC is run by a physician (GP), several health technicians, and a general administrator. Similarly distributed urban Health Posts (HP) and Urban Comprehensive Health Centers (UCHC) have been established in urban areas. HPs are staffed by community health worker “Moragheb-e-salamat” and health volunteer women from the same community and UCHCs are staffed by a physician and health technicians) which offer public health services similar to those offered by the RCHCs [9]. The whole network is supervised and administered through District Health Centers (DHC) which are mainly concerned with the management and coordination of the activities of rural and urban health centers, cooperation with more specialized district hospitals and other public medical or paramedical establishments, follow up of cases referred for secondary care from lower levels, and organization of health education activities in the district (Fig. 1).

### Introducing PHC measurement conceptual framework proposed by WHO

**Fig. 2:**
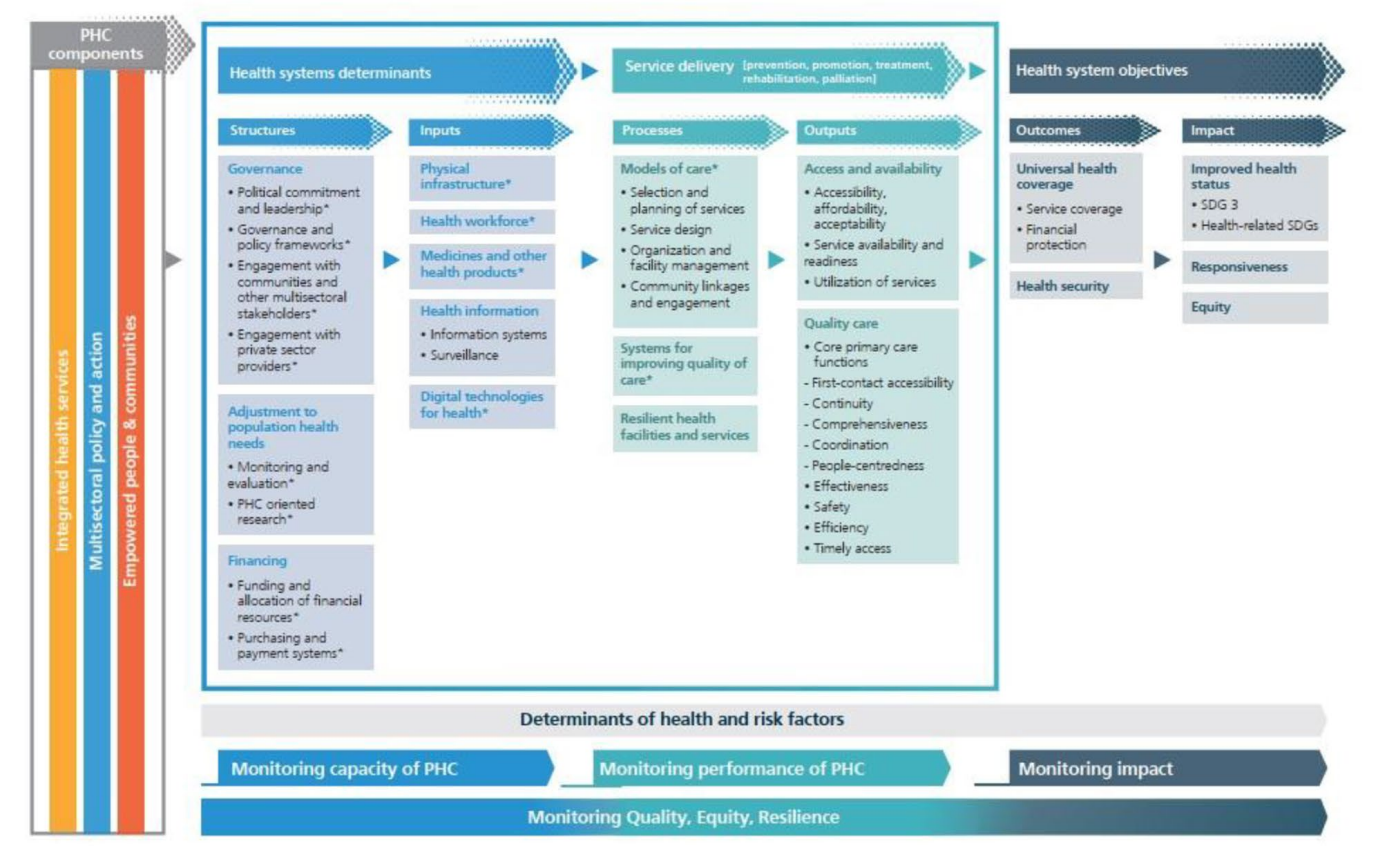
PHC monitoring conceptual framework. (Source: WHO website) [11]

The PHCMC framework was provided by WHO to continuously strengthen PHC and support member states to assess, track and monitor PHC performance improvement. This framework was developed based on and supports the levers of the operational framework introduced by PHCPI. The PHCMCF was organized in three ways, by results chain domain: structures, inputs, processes, outputs, outcomes, and impact; by PHC domain to support PHC orientation of health systems, and by PHC monitoring dimensions [11]. About 121 KPIs (2018) and 89 KPIs (2022) were introduced by WHO to assess each of these framework levers [7, 11]. These KPIs and framework have been developed based on the technical review, consultation with countries, and PHC academics and experts (Fig. 2).

### Step 1: review and adaptation of WHO’s PHC monitoring framework

To adjust KPIs and WHO’s PHC monitoring framework according to Iran’s sub-nation PHC system capacities, the National Experts Advisory Committee (NEAC) was created. This committee included 5 experts from the PHC deputy of the Ministry of Health and Medical Education (MOHME) and 5 PHC experts from the sub-national level in the field of health economics and health system financing, quality assessment, and improvement, health system management, and health systems performance assessment. Sixteen meetings of the NEAC were conducted to review KPIs provided by the WHO.

A special working group was established for each dimension of the framework and a director appointed for each. The meetings were organized by the working group director and with the participation of experts related to the dimension. The KPIs were reviewed, revised, and screened based on two criteria: the ability to collect the KPIs at the sub-national level and the relevance of the KPIs to the main function of the PHC. The working group director synthesized and summarized the comment and suggestions of the experts and made necessary editing to the KPIs.

The revision of the KPI included a change in age group, a change in the usage of the KPI (from national to the provincial level), and a change in the number of visits (for instance; increasing the number of prenatal care visits from 4 times to 6 times).

Some KPIs related to health system financing and management were removed due to not being suitable for the sub-national level. Metadata (Appendix 1) was developed for all selected KPIs, including rationality, related department, KPIs level, numerator statement, data source, denominator statement, target, data reported as, frequency of measurement, formula, and references.

### Step 2: literature review

An overview [12] was conducted using Scopus and PubMed databases to identify studies related to performance assessment indicators in PHC. The initial search keywords were the following: performance assessment, performance measurement, quality assessment, performance indicator, quality indicators, PHC, primary health care and primary care. These searches produced between the years 2000 and 2022. Studies that focused on specific populations such as the elderly and child or specific diseases such as diabetes and hypertension were excluded. Each study was reviewed and KPIs extracted. The research team reviewed selected KPIs and prepare a preliminary list of suitable KPIs.

### Step 3: series of expert panel meeting

To introduce alternative KPIs suitable for the sub-national level and fill the gaps that were created after removing the KPIs in some dimensions of the framework, six-panel sessions were held with the participation of seven experts. The criteria for selecting expert panel members included having a relevant academic degree (such as public health, epidemiology, health care service management, medicine), have at least 5 years of work experience in the PHC system, and having worked as an executive director in the PHC system at least at the district level. Experts were selected using a purposeful sampling method. Expert panel members including, two health management specialists with 18 years of management experience at provincial and national levels, two health economists with five years of management experience at county and provincial levels, one psychiatrist with 20 years of management experience at the provincial level, two experts with 15 years of work experience in statistics and analysis of KPIs.

At the beginning of the meeting, the panel leader introduced the study purpose and the previous steps of study to the participants. Then, the list of KPIs extracted from the literature (KPIs Titles) was provided to the experts. The panel leader asked the experts to select a number of KPIs that are suitable for the Iran’s PHC system and are applicable for the sub-national level. In addition to the KPIs mentioned in the initial list, a number of KPIs were also suggested by the experts. All the selected KPIs from the initial list along with the KPIs suggested by the experts were summarized by the panel leader and the KPIs were listed based on the highest frequency. The KPIs that were confirmed by more than half of the members were considered as the list of alternative KPIs for the selection through Delphi technique.

### Step 4: modified Delphi technique

The modified Delphi technique was used to choose introduced alternative KPIs that prepared by expert panel and reach a consensus on the KPIs. Selecting Delphi members was based on the same inclusion criteria as selecting expert panel members. The online modified Delphi were conducted by 10 experts. The participants in the modified Delphi survey included the director quarter of the health network management center and other related staff in the MOHME. The experts assigned an independent score ranging from 1 to 5 to each of the KPIs in three criteria included: importance, measurability, and relevance. the average scores of the KPIs were calculated based on the three criteria and adjusted to a scale of 100. The KPIs with a final mean score of more than 70 were selected; those with a mean score of 40 to 70 were sent to the second round, and the KPIs with a mean score of less than 40 were excluded.

### Step 5: final selection and development of framework

In the last step, three panel sessions were conducted for the final review of the set of KPIs selected in the previous steps, as well as their classification based on the dimensions and levers of PHC performance measurement and develop an Iranian Sub-national PHCM framework. In the first panel sessions (with the participation of 8 experts), the selected KPIs of the Delphi survey were reviewed according to 3 criteria as follows: (A) Relevance to national PHC main function, (B) Maximum coverage of current PHC processes, and (C) The possibility of interventions to improve the KPIs.

In the next two sessions (with the participation of 6 experts), each of the selected KPIs were categorized in related dimension in the conceptual framework. The final set of KPIs based on data collection sources classified into three groups:

A. Based on the PHC routine information systems;
B. Based on the facility surveys;
C. Based on the population (household) surveys.

## Results

**Fig. 3:**
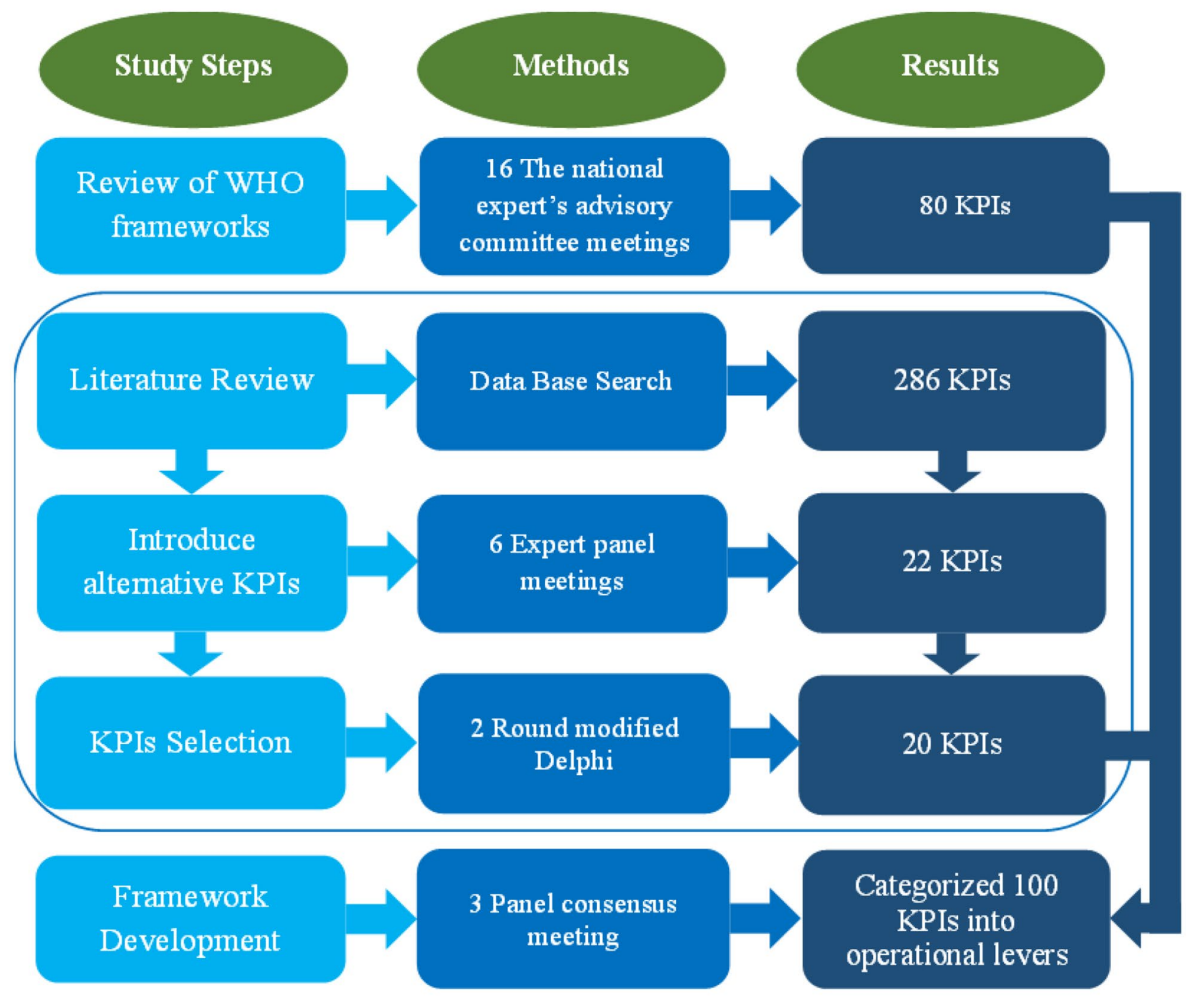
Iranian Sub-national Primary Health Care Measurement Framework Development Flow

KPIs for the measurement of Iranian sub-national PHC were reviewed and selected in five main steps. In the first step, 80 KPIs were selected from a list of WHO-recommended KPIs. Based on the literature review, 286 KPIs were identified in the second step. Among 286 KPIs, the expert panel chose 22 alternative KPIs. Throughout two rounds of modified Delphi, these KPIs were reviewed, and two of the 22 KPIs were removed. Finally, 100 KPIs were selected to develop the Iranian Sub-national PHCM framework (Fig. 3) (Appendix 2).

### Review of WHO-recommended KPIs for the PHC measurement

Among WHO-recommended KPIs (121 KPIs (2018) and 89 KPIs (2022), 80 KPIs were chosen by NEAC. KPIs were removed at this step for one of three reasons: the complete infrastructure establishment of KPIs in Iran’s PHC system, the completeness of the KPIs progress, and the lack of priority of the KPIs in Iran’s PHC system.

### Literature review

Totally, 745 KPIs were identified from 19 studies. In the initial screening, 248 KPIs were removed due to duplication. Remained KPIs were reviewed by research team based on the study object. From 496 KPIs, 47 KPIs were merged due to similarity, and 163 KPIs were removed due to lack of relevance to the study object. Finally, 286 KPIs were selected and proposed to an expert panel.

### Expert panel and Delphi survey

An expert’s panel meetings were held to review KPIs that extracted from literature and introduce alternative KPIs. In addition to the literature results, the list of Iranian PHC quality assessment indicators, national documents related to PHC, and other related documents were also reviewed by an expert panel. Based on the results of experts’ meetings, 22 KPIs were selected in accordance with the local conditions of Iran’s PHC and the sub-national level. Then, the selected KPIs were reviewed through two rounds of modified Delphi by national-level experts. Finally, 20 KPIs (out of 22) were scored higher than 70 and selected as final alternative KPIs. The full list of alternative KPIs and the average score for each KPI from the modified Delphi survey is shown in Table 1.

**Table 1:** List of alternative key performance indicators with Results from the modified Delphi Survey

	Key Performance Indicators	Relevance	Importance	Feasibility	Total *
1	Percentage of the Provincial High Council for Health and Food Safety Decisions that have been implemented	93.13	95	95	94.37
2	Complaint response rate during the first 72 h	87.5	82.5	85	85
3	Percentage of health facilities with an annual operational plan.	85	80	87.5	84.17
4	Percentage of households with a health volunteer	82.5	95	85	87.50
5	A number of applied research projects implemented in the district health network.	87.5	87.5	70	81.67
6	PHC expenditure as % of total Sub-national health expenditure	90	95	83.33	89.44
7	Mental health expenditure as % of total Sub-national PHC expenditure	82.5	72.5	70	75
8	Medicine/drugs expenditure as % of total Sub-national PHC expenditure	85.63	76.88	80	80.83
9	Payment period for HWs working extra hours in the healthcare facility	87.5	84.38	73.13	81.67
10	Implementation of the managers’ capacity building program, regularly	85	97.5	80	87.5
11	Percentage of catchment population who received at least one basic visit	92.5	98.13	85	91.88
12	Customer Satisfaction Rate	98.13	97.5	95	96.88
13	Health Worker Satisfaction Rate	92.5	93.13	90	91.88
14	Percentage of HWs trained on occupational health safety and risk management in the healthcare facility	93.13	88.75	80	87.29
15	Percentage compliance with Hand Hygiene guidelines	86.25	78.75	76.25	80.42
16	Percentage of trained HWs on Infection Prevention Control	93.13	86.88	83.75	87.92
17	Percentage of health facilities that have a fire safety and building evacuation program.	85.63	83.75	83.75	84.38
18	Average waiting time (min) at PHC facilities	86.25	88.13	73.75	82.71
19	Percentage of appropriate (upward) referrals during the last 6 months (by specific conditions) with appropriate feedback	97.5	96.25	63.75	85.83
20	Patients’ perceptions of PHC system responsiveness	85.63	89.38	65.63	80.21

*Average

Relevance: The indicator is related to the main processes at the provincial and district levels

Importance: The indicator reflects important aspects of PHC system performance at the provincial and district levels

Feasibility: There is the necessary infrastructure and conditions to measure the indicator

### Developing Iranian sub-national PHCM framework

The PHCMC framework introduced by WHO (Fig. 1) has three main components: health systems determinants, Service Delivery, and Health system objectives at six results chain domains, including Structure, Input, Process, Output, Outcome, and Impact. The operational levers and core strategic levers are located inside these results chain domains. Panel consensus meetings were held to review the KPIs and classify the KPIs among the levers.

The following was the distribution of KPIs for measurement of Iran’s sub-national PHC system based on the results chain domains: 16% (16 KPIs) were related to the structure, 20% (20 KPIs) the input, 9% (9 KPIs) the process, 24% (24 KPIs) the output, 9% (9 KPIs) the outcome and 22% (22 KPIs) the impact (Table 2). Most of the KPIs introduced by the expert panel are related to the governance and financing levers at the structural level. Because the KPIs introduced by the WHO for these levers were at the macro and national levels and did not reflect the trend of change at the sub-national level and also it was not possible to measure it at the sub-national level, so for those KPIs, alternatives were introduced and selected. Also, due to the importance of health workers and patient safety and the lack of interventions in the Iranian PHC system about them, the safety lever (8 KPIs) was further emphasized in the Iranian sub-national PHCM framework.

**Table 2:** Number of Iranian sub-national PHC Key Performance Indicators based on PHC levers

PHC domain	results chain domain	Operational levers	Core levers	KPIs Selected From WHO Framework	KPIs Suggested by Expert Panel
**Health systems determinants**	Structure	Governance	Political commitment and leadership	0	2
			Governance and policy frameworks	0	1
			Engagement with communities and other multisectoral stakeholders	1	1
			Engagement with private sector providers	1	0
		Adjustment to population health needs	Monitoring and evaluation	2	0
			PHC oriented research	1	1
		Financing	Funding and allocation of financial resources	1	3
			Purchasing and payment systems	0	1
	Input	Physical Infrastructure	-	4	0
		Health Workforce	-	6	0
		Medicines and other health products	-	2	0
		Health Information Systems	Information systems	6	0
			Surveillance	1	0
		Digital technologies for health	-	1	0
**Service delivery**	Process	Model of Care	Selection and planning of services	2	0
			Service design	1	0
			Organization and facility management	0	1
			Community linkages and engagement	2	0
		Systems for improving the quality of care	-	2	0
		Resilient health facilities and services	-	1	0
	Output	Access and availability	Accessibility, affordability, acceptability	2	0
			Service availability and readiness	2	0
			Utilization of services	0	1
		Quality care	People-centeredness	0	2
			Effectiveness	6	0
			Safety	4	4
			Efficiency	1	0
			Timely access	0	2
**Health system objectives**	Outcome	Universal health coverage	Service coverage	5	0
			Financial protection	1	0
		Health security	-	3	0
	Impact	Improved health status	Good Health and Well-being	7	0
			Health-related SDGs	12	0
		Responsiveness	-	0	1
		Equity	-	3	0
Number of KPIs				80	20

## Discussion

Iranian sub-national PHCMF was developed in five main steps, using valid and scientific methods (review of WHO framework, literature review, national document review, national expert meeting, and modified Delphi technique). Iranian sub-national PHCM framework was finalized with 100 KPIs in three components including Health systems determinants, Service Delivery, and Health system objectives at six results chain domains including Structure, Input, Process, Output, Outcome, and Impact.

One of the most important challenges facing the WHO in implementing measures to assess the performance of PHC in LMICs is the lack of information infrastructure for data collection [13, 14]. Therefore, most countries use local measures to assess their PHC system [14, 15]. Iran as a LMICs, according to the experiences of cooperation with the WHO in developing the quality assessment framework in PHC, and implementing the PHCMI at the national level in recent years, has been able to create health information infrastructure and adequate resources and data to assess PHC performance [4]. Using these experiences, most of the KPIs of Iranian sub-national PHCMF (80 KPIs) are among the KPIs proposed by the WHO, and only in some of the levers, alternative KPIs were suggested by experts.

According to the results chain domain [16], the majority of the selected KPIs (55%) are related to the output, outcome, and impact which focused on UHC and health system objectives. Due to PHC is a strategy for strengthening health systems to accelerate progress toward UHC [17], focusing on KPIs at these levels can accurately assess progress toward UHC and highlight real weaknesses and challenges. Measurement and tracking of these KPIs could lead to provide effective services in responding to people’s needs.

The structure of Iran’s PHC system is centralized, and health laws and regulations are formulated by the MOHME and communicated to sub-national levels [18]. In this regard, PHC expenditures are allocated by the MOHME as a global budget to the sub-national level, and the provinces cannot decide on financing independently. The KPIs proposed by the WHO, including the presence of UHC legislation inclusive of PHC and total PHC spending per capita, could not reflect the funding differences between the provinces and assess their status in governance and financing levers. Therefore, the alternative KPIs, including the percentage of the Provincial High Council for Health and Food Safety Decisions that have been implemented, percentage of health facilities with an annual operational plan, charity as % of total PHC expenditure, and PHC expenditure as % of total Sub-national health expenditure, were selected for this lever.

Satisfaction of health workers and customers is one of the most important components in quality assessment [19]. The low satisfaction level of health workers has a major impact on leaving the job and performance [20]. Dissatisfied health workers are more likely to have errors and high satisfaction of health workers leads to reduced healthcare costs [21]. Previous studies in Iran have shown that the level of health workers and customer satisfaction is low [22]. Unfortunately, the KPIs proposed by the WHO were neglected. In order to fill this gap and the need to improve health workers and customer satisfaction, experts selected satisfaction-related KPIs for the Iranian framework.

Patient and health workers’ safety are fundamental concepts for achieving the Sustainable Development Goal. the WHO Thirteenth General Program of Work, the Triple Billion targets of WHO, and the strategic vision of EMRO have all prioritized health workers’ safety [23, 24]. health workers as the frontline defenders during the Covid-19 pandemic have been at high risk of contracting the infection [25–27]. health workers and patient safety are one of the topics that are less considered in Iran’s PHC system. In order to complete the KPIs proposed by the WHO, four KPIs related to patient and health worker safety were added to the Iranian sub-national PHCM framework.

### Applicability of this study

The timeframe of data collection for each KPIs is predicted in their meta data, and it ranges from three months to one year. The KPIs information will be reported according to the decision-making levels. The information of all KPIs is reported to senior managers and policymakers, and for responsible unite, only those indicators relevant to that unite are reported.

Iranian sub-national PHCM framework could be used as a comprehensive tool to track and improve PHC performance. Also, this framework could be used to compare provinces and districts performances. According to this framework, managers and decision-makers at the sub-national level can translate local goals into action. The measurement of PHC performance through this framework is an opportunity to identify the weaknesses and challenges and developed suitable interventions to address them. Furthermore, using this PHCM framework in different countries, especially LMICs, will provide an opportunity to share successful experiences between similar contexts.

### Limitation

The lack of available data for some KPIs is one of the most important limitations. There is currently no data available for KPIs that require household surveys. In order to provide a comprehensive framework that covers all functional components of PHC, it was necessary to introduce KPIs for all of these components. Also, the purpose of including KPIs that require household surveys was to expand the information platforms of the PHC system to cover these types of KPIs.

Another limitation of the study; was the inclusion of consumer satisfaction indicator in the framework. This indicator cannot accurately reflect consumer expectations. Because more vulnerable populations have lower expectations for their healthcare and are consequently satisfied by poorer quality than well-off groups.

## Conclusion

The Iranian sub-national PHCM framework was developed in accordance with a suggested framework by WHO, relevant global and regional evidence, and with the participation of PHC national experts and managers. This framework, as a comprehensive and scientific tool, can play a vital role in translating goals to action plans and continuous quality improvement in the PHC system. As a result, it is recommended that this framework and suggested KPIs be used for quantitative and qualitative measurement of the Iranian PHC system performance, including the resources and infrastructure required to provide services, human resource management, quality of care, patient and health worker safety and other aspect PHC performance.

## Electronic supplementary material

Below is the link to the electronic supplementary material.

Supplementary Material 1

Supplementary Material 2

## Data Availability

All data generated or analyzed during this study are included in this published article.

## List of Abbreviations

EMRO: Eastern Mediterranean Regional Office
KPIs: Key Performance Indicators
LMICs: Low- and Middle-Income Countries
NEAC: National Experts Advisory Committee
PHC: Primary Health Care
PHCMF: Primary Health Care Measurement Framework
PHCMI: Primary Health Care Measurement Initiative
WHO: World Health Organization
