# Supplementary material for: Developing Iranian sub-national primary health care measurement framework: a mixed-method study
Source: Arch Public Health. 2023 Jun 1;81:98. doi: 10.1186/s13690-023-01108-0 (PMC10233173; doi:10.1186/s13690-023-01108-0)
Supplement: Supplementary file 1 — Supplementary Material 1 [file 13690_2023_1108_MOESM1_ESM.docx]

# **Final Set of Indicators for Sub-National PHCMI**

The following is an overview of how to prepare the metadata of an indicator. In this fine meta data of 82 KPIs were introduced as a sample.

**Inputs**

**A-Human Resources**

1. **% Health workforce in primary care (by occupation)**

**Responsible Unit:** Health Network management

**Type:** Structure

**Source (s):** Routine System

**Type of Reporting:** %

**Interval:** Annually

**References:**

- World Health Organization. National health workforce accounts: a handbook; 2017
- Dussault G, Kawar R, Castro Lopes S, Campbell J. Building the primary health care workforce of the 21st century. Background paper to the Global Conference on Primary Health Care: From Alma-Ata towards Universal Health Coverage and the Sustainable Development Goals. Geneva: World Health Organization; 2018.
- Health workforce requirements for universal health coverage and the sustainable development goals. Human Resources for Health Observer Series No. 17. Geneva: World Health Organization; 2017

1. **% Primary care workforce specialized in family practice (by occupation)**

**Responsible Unit:** Health Network Management

**Type:** Structure

**Source (s):** Routine System

**Type of Reporting:** %

**Interval:** Annually

**References:**

- Health workforce requirements for universal health coverage and the sustainable development goals. Human Resources for Health Observer Series No. 17. Geneva: World Health Organization; 2017
- World Health Organization. National health workforce accounts: a handbook. 2017
- World Health Organization. National health workforce accounts: implementation guide. 2018
- World Health Organization. National Health Workforce Accounts: Better data and evidence on health workforce.2019

1. **Proportion of HWF in PHC have received minimum continuous professional education according to national requirements in the last year**

**Responsible Unit:** Human Resources Office

**Type:** Structure

**Source (s):** Routine System

**Type of Reporting:** %

**Interval:** Annually

**References:**

- World Health Organization. National health workforce accounts: a handbook. 2017
- WHO. National health workforce accounts: implementation guide. 2018
- World Health Organization. National Health Workforce Accounts: Better data and evidence on health workforce.2019
- Global Strategy on Human Resources for Health: Workforce 2030. Geneva: World Health Organization; 2016

1. **Vacancy rate in PHC (all levels)**

**Responsible Unit:** Human Resources Office

**Type:** Structure

**Source (s):** Routine System

**Type of Reporting:** %

**Interval:** Annually

**References:**

- World Health Organization. National health workforce accounts: a handbook. 2017
- World Health Organization. National health workforce accounts: implementation guide. 2018
- World Health Organization. National Health Workforce Accounts: Better data and evidence on health workforce. 2019

1. **Density of PHC by occupation (N/10,000 population)**

**Responsible Unit:** Health Network Management

**Type:** Structure

**Source (s):** Routine System

**Type of Reporting:** %

**Interval:** Annually

**References:**

- World Health Organization. National health workforce accounts: a handbook. 2017
- World Health Organization. National health workforce accounts: implementation guide. 2018
- World Health Organization. National Health Workforce Accounts: Better data and evidence on health workforce. 2019

**B-Information System**

1. **% births registered**

**Responsible Unit:** Health Network Management

**Type:** Structure

**Source (s):** Routine System (Iman), Sabt-e-Ahval

**Type of Reporting:** %

**Interval:** Annually

**References:**

- Fagernäs S, Odame J. Birth registration and access to health care: an assessment of Ghana's campaign success. Bulletin of the World Health Organization. 2013; 91:459-64.
- United Nations Children’s Fund [Internet]. Childinfo: percentage of children under five who were registered at the moment of the survey. New York: UNICEF; 2013.
- Bhutta ZA, Chopra M, Axelson H, Berman P, Boerma T, Bryce J, et al., et al. Countdown to 2015-decade report (2000–10): taking stock of maternal, newborn, and child survival. *Lancet* 2010; 375: 2032-44
- Setel PW, Macfarlane SB, Szreter S, Mikkelsen L, Jha P, Stout S, et al., Monitoring of Vital Events, et al. A scandal of invisibility: making everyone count by counting everyone. *Lancet* 2007; 370: 1569-77

1. **% deaths registered**

**Responsible Unit:** Health Network Management

**Type:** Structure

**Source (s):** Routine System (Iman), Sabt-e-Ahval

**Type of Reporting:** %

**Interval:** Annually

**References:**

- Completeness and coverage of death registration data: Health statistics and information systems, World Health Organization.
- Adair T, Lopez AD. Estimating the completeness of death registration: an empirical method. PloS one. 2018 May 30;13(5): 0197047.

1. **Explicit adoption of a set of PHC indicators for Monitoring and Evaluation (all levels)**

**Responsible Unit:** Monitoring and Evaluation Office

**Type:** Process

**Source (s):** Routine System Records

**Type of Reporting:** Qualitative (Yes/No)

**Interval:** Annually

**References:**

- Murray CJ, Frenk J, World Health Organization. A WHO framework for health system performance assessment.
- Fiume R, Ramazzotti G, Faenza I, Piazzi M, Bavelloni A, Billi AM, Cocco L. Nuclear PLCs affect insulin secretion by targeting PPARγ in pancreatic β cells. The FASEB Journal. 2012 Jan;26(1):203-10.

1. **Inclusion of section on PHC performance in annual health sector reporting (all levels)**

**Responsible Unit:** Monitoring and Evaluation Office

**Type:** Outcome

**Source (s):** Routine System Records

**Type of Reporting:** Qualitative (Yes/No)

**Interval:** Annually

**References:**

- The Primary Health Care Performance Initiative. Strong primary health care saves live in times of crisis and calm. 2018. https://www.phcperformanceinitiative.org
- Campbell SM, Braspenning JA, Hutchinson A, Marshall M. Research methods used in developing and applying quality indicators in primary care. Quality and Safety in Health Care. 2002 Dec 1;11(4):358-64.

1. **% private and public sector PHC that reports performance data**

**Responsible Unit:** Health Network Management

**Type:** Outcome

**Source (s):** Routine System (Iman), Sabt-e-Ahval

**Type of Reporting:** %

**Interval:** Annually

**References:**

- Fekri O, Klazinga N. Health system performance assessment in the WHO European Region: which domains and indicators have been used by Member States for its measurement? World Health Organization; 2018 Apr 24.
- Tabrizi JS, Azami-Aghdash S, Gharaee H. Public-Private Partnership Policy in Primary Health Care: A Scoping Review. Journal of primary care & community health. 2020 Aug; 11:2150132720943769.

1. **Presence and use of unique identifiers at facility**

**Responsible Unit:** IT Office

**Type:** Structure

**Source (s):** Routine System

**Type of Reporting:** Qualitative (Yes/No)

**Interval:** Annually

**References:**

- WHO, JCI Patient Safety Solutions, volume 1, solution 2, May 2007, available at: <http://www.who.int/patientsafety/solutions/patientsafety/PS-Solution2.pdf>
- Australian commission on Safety and Quality in Healthcare. Patient Identification; available at: http://www.safetyandquality.gov.au/our-work/patient-identification.

1. **% of patients who get registered by PHC facilities**

**Responsible Unit:** IT Office

**Type:** Structure

**Source (s):** Routine System

**Type of Reporting:** %

**Interval:** Annually

**References:**

- - ERPHO (2003) INphoRM 2: catchment areas and populations. Available at: http: [www.erpho.org.uk/viewResource.aspx?id=9480](http://www.erpho.org.uk/viewResource.aspx?id=9480)
  - CACI (n.d) ACORN segmentation. Available at: [www.caci.co.uk/acorn-classification.aspx](http://www.caci.co.uk/acorn-classification.aspx)
  - J Cullinana, S Hynesa and C O’Donoghueb “Estimating Catchment Area Population Indicators Using Network Analysis”.

1. **Presence of a comprehensive individual patient/family record**

**Responsible Unit:** IT Office

**Type:** Structure

**Source (s):** Routine System

**Type of Reporting:** Qualitative (Yes/No)

**Interval:** Annually

**References:**

- WHO. Electronic Health Records, A Manual for Developing Countries. 2006
- King J, Patel V, Jamoom EW, Furukawa MF. Clinical benefits of electronic health record use: national findings. Health services research. 2014 Feb;49(1pt2):392-404.
- Kalra, D. (2006). Electronic health record standards

1. **Is there a functioning eHIS in the province/city?**

**Responsible Unit:** Health Network Management

**Type:** Structure

**Source (s):** Routine System

**Type of Reporting:** %

**Interval:** Annually

**References:**

- Tomasi E, Facchini LA, Maia MD. Health information technology in primary health care in developing countries: a literature review. Bulletin of the World Health Organization. 2004; 82:867-74.
- Yazdi-Feyzabadi V, Emami M, Mehrolhassani MH. Health information system in primary health care: the challenges and barriers from local providers’ perspective of an area in Iran. International journal of preventive medicine. 2015; 6

**C- Infrastructures**

1. **% population that would have to travel more than 5 km/1 hour to arrive at PHC facility**

**Responsible Unit:** Health Network Management

**Type:** Structure

**Source (s):** Routine System

**Type of Reporting:** %

**Interval:** Annually

**References:**

- Zinszer K, Charland K, Kigozi R, Dorsey G, Kamya MR, Buckeridge DL. Determining health-care facility catchment areas in Uganda using data on malaria-related visits. Bulletin of the World Health Organization. 2014 Jan 10; 92:178-86.
- Jones S, Wardlaw J, Crouch S, Carolan M. Modelling catchment areas for secondary care providers: a case study. Health Care Manag Sci 2011; 14: 253-61 [http://dx.doi.org/10.1007/s10729-011-9154-y](https://dx.doi.org/10.1007/s10729-011-9154-y) pmid: [21455707](https://www.ncbi.nlm.nih.gov/sites/entrez?cmd=Retrieve&db=PubMed&list_uids=21455707&dopt=Abstract).
- Boscoe FP, Henry KA, Zdeb MS. A nationwide comparison of driving distance versus straight-line distance to hospitals. Prof Geogr 2012; 64: 188-96

1. **% PHC facilities with adequate WASH**

**Responsible Unit:** Health Network Management

**Type:** Structure

**Source (s):** Routine System

**Type of Reporting:** %

**Interval:** Annually

**References:**

- WHO (2008). Essential environmental health standards in health care. Geneva: World Health Organization.
- WHO (2014). UN-Water global analysis and assessment of sanitation and drinking-water (GLAAS) 2014 report. Investing in water and sanitation: increasing access, reducing in inequalities. Geneva: World Health Organization.
- WHO/UNICEF (2013). End preventable deaths: Global action plan for prevention and control of pneumonia and diarrhea (GAPPD). Geneva: World Health Organization.

1. **Room with auditory and visual privacy for patient consultations**

**Responsible Unit:** Health Network Management

**Type:** Structure

**Source (s):** Facility Survey

**Type of Reporting:** %

**Interval:** Annually

**References:**

- WHO. Patient Safety and Rights: Developing tools to support consumer health literacy. 2010
- Terry NP. Protecting patient privacy in the age of big data. UMKC L. Rev. 2012; 81:385. 2015; 6.

1. **Communication equipment (phone or SW radio)**

**Responsible Unit:** Health Network Management

**Type:** Structure

**Source (s):** Routine System

**Type of Reporting:** %

**Interval:** Annually

**References:**

- World Health Organization. Primary health care: transforming vision into action: operational framework. World Health Organization; 2018
- Global diffusion of eHealth: Making universal health coverage achievable. Geneva: World Health Organization; 2016

1. **Facility has access to computer with email/internet access**

**Responsible Unit:** Health Network Management

**Type:** Structure

**Source (s):** Routine System

**Type of Reporting:** %

**Interval:** Annually

**References:**

- World Health Organization. Primary health care: transforming vision into action: operational framework. World Health Organization; 2018.
- Global diffusion of eHealth: Making universal health coverage achievable. Geneva: World Health Organization; 2016

1. **Standard precautions for infection prevention**

**Responsible Unit:** Health Network Management

**Type:** Structure

**Source (s):** Facility Survey

**Type of Reporting:** %

**Interval:** Annually

**References:**

- WHO. Guidelines on core components of infection prevention and control programs at the national and acute health care facility level. 2016

**D-Medicines**

1. **PHC EML list correlated to package of services delivered in PHC**

**Responsible Unit:** Health Network Management

**Type:** Outcome

**Source (s):** Facility Survey

**Type of Reporting:** %

**Interval:** Annually

**References:**

- World Health Organization. Regional Office for South-East Asia. Essential drugs for primary health care. WHO Regional Office for South-East Asia.

1. **Proportion of facilities in which essential medicines are available (no stock outs in X time frame)**

**Responsible Unit:** Health Network Management

**Type:** Outcome

**Source (s):** Facility Survey

**Type of Reporting:** %

**Interval:** Annually

**References:**

- PHCPI: Service Delivery Indicators (SDI). set of health indicators that examine health workers’ effort and ability, as well as the availability of key inputs and resources that contribute to the functioning of a health facility

**Process Indicators**

**A-Model of health care provision**

1. **% of PHC cases referred to secondary care**

**Responsible Unit:** Health Network Management

**Type:** Outcome

**Source (s):** Routine System

**Type of Reporting:** %

**Interval:** Annually

**References:**

- Paulo De Marco, Caroline Dain, Trevor Lockwood, Martin Roland. How Valuable Is Feedback of Information On Hospital Referral Patterns? BMJ Volume 307 4 December 1993
- Jamal S. Jarallah, Mrcgp. Referral from Primary Care to Hospitals in Saudi Arabia: Quality of Referral Letters and Feedback Reports. J Family Community Med. 1998 Jul-Dec; 5(2): 15–22

1. **Annual outpatient department utilization rates per capita**

**Responsible Unit:** Health Network Management

**Type:** Outcome

**Source (s):** Utilization Health Survey

**Type of Reporting:** Per capita (ratio)

**Interval:** Annually

**References:**

- Hosseinpoor AR, Naghavi M, Alavian SM, Speybroeck N, Jamshidi H, Vega J. Determinants of seeking needed outpatient care in Iran: results from a national health services utilization survey. *Arch Iran Med.* 2007; 10(4): 439 -45
- Ravangard R, Hatam N, Teimourizad A, Jafari A. Factors affecting the technical efficiency of health systems: A case study of Economic Cooperation Organization (ECO) countries (2004–10). *International journal of health policy and management.* 2014; 3(2): 63 -69

1. **% of PHC facilities that can provide mental health services**

**Responsible Unit:** Mental Health Office

**Type:** Structure

**Source (s):** Routine System

**Type of Reporting:** %

**Interval:** Annually

**References:**

- Integrating mental health services into primary health care. Geneva, World Health Organization, 2007 ; Available at : <http://www.who.int/mental_health/policy/services/3_MHintoPHC_Infosheet.pdf>

1. **Number of consultations per health worker (physician, nurse, etc.) per day**

**Responsible Unit:** Health Network Management

**Type:** Outcome

**Source (s):** Household Survey (Utilization)

**Type of Reporting:** Per capita (ratio)

**Interval:** Annually

**References:**

- World Health Organization. Primary health care: transforming vision into action: operational framework. World Health Organization; 2018
- Liu S, Wang H, Gao B, Deng Z. Doctors’ Provision of Online Health Consultation Service and Patient Review Valence: Evidence from a Quasi-Experiment. Information & Management. 2020 Aug 13:103360

**B-Management/Quality Improvement indicators**

1. **Proportion of facilities with up-to-date performance reports in the last 6 months to 1 year**

**Responsible Unit:** Health Network Management

**Type:** Process

**Source (s):** Routine System

**Type of Reporting:** Ratio

**Interval:** Annually

**References:**

- Fekri O, Klazinga N. Health system performance assessment in the WHO European Region: which domains and indicators have been used by Member States for its measurement? World Health Organization; 2018 Apr 24.
- Murray CJ, Frenk J. A framework for assessing the performance of health systems. Bulletin of the world Health Organization. 2000; 78:717-31

1. **% PC facilities with systems to support quality improvement**

**Responsible Unit:** Health Network Management

**Type:** Process

**Source (s):** Facility Survey

**Type of Reporting:** %

**Interval:** Annually

**References:**

- World Health Organization. A vision for primary health care in the 21st century: towards universal health coverage and the Sustainable Development Goals. World Health Organization; 2018.
- Rezapour R, Tabrizi JS, Farahbakhsh M, Saadati M, Abdolahi HM. Developing Iranian primary health care quality framework: a national study. BMC public health. 2019 Dec 1;19(1):911.

**E-Quality Processes (Patient Survey) indicators**

1. **% PC facilities that monitor patient experience**

**.**

**Responsible Unit:** Health Network Management

**Type:** Process

**Source (s):** Facility Survey

**Type of Reporting:** %

**Interval:** Annually

**References:**

- Waghorn A, McKee M. Understanding patients' views ofa surgical outpatient clinic. J. Eval. Clin. Pract 2000; 6:273-9.
- Jenkins K, Grady D, Wong J, Correa R, Armanious S, Chung F. Post-operative recovery: Day surgery patients' preferences. British Journal of Anaesthesia 2001; 86:272-4
- Development and Validation of Scales to Measure Patient Satisfaction with Medical Care Services (1976) Ware, JE, Snyder, MK, and Wright, WR. Vol I, Part B: Results Regarding Scales Constructed from the Patient Satisfaction Questionnaire and Measures of Other Health Care Perceptions. (NTIS Publication No. PB 288-329). Springfield, VA. National Technical Information Service

**Output indicators**

**A-Effective Coverage/Quality of Care indicators**

1. **Number of adverse events reported (immunization/medication)**

**Responsible Unit:** Non-Communicable Diseases Office

**Type:** Process

**Source (s):** Routine System

**Type of Reporting:** %

**Interval:** Annually

**References:**

• Gandhi TK, Weingart, SN, Borus J, Seger AC, Peterson J, Burdick E, et al. Adverse Drug Events in Ambulatory Care. New England Journal of Medicine [Internet]. April 17, 2003 348(16): 1556.Available from: http://www.nejm.org/doi/full/10.1056/NEJMsa020703

• World Health Organization, WHO Patient Safety Assessing and tackling patient harm: a methodological guide for data-poor hospitals. 2010

1. **% of PHC prescriptions that include antibiotics in out-patient clinics**

**Responsible Unit:** Drug Office

**Type:** Outcome

**Source (s):** Routine System

**Type of Reporting:** %

**Interval:** Annually

**References:**

- Al-Niemat, S. I., Bloukh, D. T., Al-Harasis, M. D., Al-Fanek, A. F., & Salah, R. K. (2008). Drug use evaluation of antibiotics prescribed in a Jordanian hospital outpatient and emergency clinics using WHO prescribing indicators. Saudi medical journal, 29(5), 743-748.
- World Health Organization. 2001. Interventions and Strategies to Improve the Use of Antimicrobials in Developing Countries: Drug Management Program. Geneva: WHO; (WHO/CDS/CSR/DSR/2001.9)

1. **% of PHC prescriptions that include injectable medicines**

**Responsible Unit:** Drug Office

**Type:** Outcome

**Source (s):** Routine System

**Type of Reporting:** %

**Interval:** Annually

**References:**

- Ayoub SW, Musalam AH, Mahadi AA. Drug utilization in primary healthcare centres in the Gaza Strip. Eastern Mediterranean Health Journal. 2017 Oct 1;23(10).
- Bairami F, Soleymani F, Rashidian A. Improving injectable medicines prescription in outpatient services: a path towards rational use of medicines in Iran. International journal of health policy and management. 2016 May;5(5):321.

1. **% of registered hypertension patients with BP <140/90 at last 2 follow up visits**

**Responsible Unit:** Non-Communicable Diseases Office

**Type:** Outcome

**Source (s):** Routine System

**Type of Reporting:** %

**Interval:** Annually

**References:**

- World Health Organization. (2007). Prevention of cardiovascular disease: guidelines for assessment and management of total cardiovascular risk. In Prevention of cardiovascular disease: guidelines for assessment and management of total cardiovascular risk. WHO.
- World Health Organization. (2007). Prevention of cardiovascular disease: pocket guidelines for assessment and management of cardiovascular risk.

1. **% of registered diabetic patients with fasting blood sugar controlled at last 2 follow up visits/A1C <7%**

**Responsible Unit:** Non-Communicable Diseases Office

**Type:** Outcome

**Source (s):** Routine System

**Type of Reporting:** %

**Interval:** Annually

**References:**

- World Health Organization. Definition, Diagnosis and Classification of Diabetes Mellitus and its Complications. Part 1: Diagnosis and Classification of Diabetes Mellitus. WHO/NCD/NCS/99.2 ed. Geneva, World Health Organization, 1999
- Kapadia and Zeitler. “Hemoglobin A1c measurement for the diagnosis of Type 2 diabetes in children” International Journal of Pediatric Endocrinology 2012, 2012:31

1. **% of registered NCD patients with 10 years cardiovascular risk recorded in past 1 year**

**Responsible Unit:** Non-Communicable Diseases Office

**Type:** Outcome

**Source (s):** Routine System

**Type of Reporting:** %

**Interval:** Annually

**References:**

- WHO. 2015 NCD Global Monitoring Framework: Indicator Definitions and Specifications. Available at: http://www.who.int/nmh/ncdtools/indicators/GMF_Indicator_Definitions_FinalNOV2014.pdf
- WHO. 2014. Package of essential non communicable disease interventions for low resource settings: Implementation tools.

1. **% of women who delivered and received at least once postnatal care within the first 40 days**

**Responsible Unit:** Population and Reproductive Health Office

**Type:** Outcome

**Source (s):** Routine System

**Type of Reporting:** %

**Interval:** Annually

**References:**

- WHO recommendations on postnatal care of the mother and newborn; available at: http://apps.who.int/iris/bitstream/10665/97603/1/9789241506649_eng.pdf
- WHO documentation: available at: http://www.who.int/maternal_child_adolescent/topics/newborn/postnatal_care/en/ http://www.who.int/maternal_child_adolescent/documents/924159084x/en/index.html.

1. **% of substance users including tobacco users in receipt of brief intervention**

**Responsible Unit:** Mental Health Office

**Type:** Outcome

**Source (s):** Routine System

**Type of Reporting:** %

**Interval:** Once every six months

**References:**

- WHO. 2003. Brief Intervention for Substance Use: A Manual for Use in Primary Care. Available at:

<http://www.who.int/substance_abuse/activities/en/Draft_Brief_Intervention_for_Substance_Use.pdf>

- WHO. 2010. The ASSIST-linked brief intervention for hazardous and harmful substance use: manual for use in primary care. Available at: http://apps.who.int/iris/bitstream/10665/44321/1/9789241599399_eng.pdf

1. **% of under 5 children that had weight and height measured in past 1 year**

**Responsible Unit:** Population and Reproductive Health Office

**Type:** Outcome

**Source (s):** Routine System

**Type of Reporting:** %

**Interval:** Annually

**References:**

- Use of growth charts for assessing and monitoring growth in Canadian infants and children: Executive summary. *Paediatr Child Health*. 2004; 9(3): 171–173

1. **Children under 5 who are stunted, wasted, overweight, obese**

**Responsible Unit:** Nutrition and Health Office

**Type:** Outcome

**Source (s):** Household Survey

**Type of Reporting:** %

**Interval:** Annually

**References:**

- Black, R.E., et al., Maternal and Child Undernutrition and Overweight in Low-income and Middle-income Countries, Lancet, vol. 382, no. 9890, 3 August 2013, pp. 427–451
- de Onis, Mercedes, et al., Comparison of the World Health Organization (WHO) Child Growth Standards and the National Center for Health Statistics/WHO International Growth Reference: Implications for child health programmes, Public Health Nutrition, vol. 9, no. 7, 2006, pp. 942–947

1. **Exclusive Breastfeeding 0-5 months (%)**

**Responsible Unit:** Population and Reproductive Health Office

**Type:** Outcome

**Source (s):** Routine System

**Type of Reporting:** %

**Interval:** Annually

**References:**

- WHO recommendations on postnatal care of the mother and newborn; available at http://apps.who.int/iris/bitstream/10665/97603/1/9789241506649_eng.pdf
- Lancet: <http://www.thelancet.com/series/breastfeeding>
- WHO websites: http://www.who.int/life-course/news/commentaries/breastfeeding-can-save-lives/en/ o <http://www.who.int/nutrition/topics/infantfeeding/en/>.

1. **Cervical cancer screening rates among women 30-59 years old**

**Responsible Unit:** Population and Reproductive Health Office

**Type:** Outcome

**Source (s):** Routine System

**Type of Reporting:** %

**Interval:** Annually

**References:**

- Kringos DS, Boerma WG, Bourgueil Y, Cartier T, Hasvold T, Hutchinson A, et al. The European primary care monitor: structure, process and outcome indicators. BMC family practice. 2010;11(1):81
- Viens LJ, Clouston S, Messina CR. Women's autonomy and cervical cancer screening in the Lesotho Demographic and Health Survey 2009. Social Science & Medicine. 2016 Feb 29; 150:23-30

1. **Vaccination of Measles2 and DTP3 (infant-under 23 months age)**

**Responsible Unit:** Communicable Diseases Office

**Type:** Outcome

**Source (s):** Routine System

**Type of Reporting:** %

**Interval:** Annually

**References:**

- Center for Global Development. Making Markets for Vaccines. http://www.who.int/intellectualproperty/news/en/SubmissionBarder1.pdf
- The Childhood Immunization Schedule and Safety: Stakeholder Concerns, Scientific Evidence, and Future Studies (2013)

**System/Structure Indicators**

1. **Existence of community-based health workforce, density**

**Responsible Unit:** Health Network Management

**Type:** Structure

**Source (s):** Routine System/ Iranian National Statistical Organization

**Type of Reporting:** %

**Interval:** Annually

**References:**

- Dussault G, Kawar R, Castro Lopes S, Campbell J. Building the primary health care workforce of the 21st century. Background paper to the Global Conference on Primary Health Care: From Alma-Ata towards Universal Health Coverage and the Sustainable Development Goals. Geneva: World Health Organization; 2018.
- Global Strategy on Human Resources for Health: Workforce 2030. Geneva: World Health Organization; 2016
- Health workforce requirements for universal health coverage and the sustainable development goals. Human Resources for Health Observer Series No. 17. Geneva: World Health Organization; 2017

**Outcome indicators**

1. **% households with adequate WASH**

**Responsible Unit:** Environmental Health Office

**Type:** Outcome

**Source (s):** Facility Survey

**Type of Reporting:** %

**Interval:** Annually

**References:**

- World Health Organization. Guidelines for drinking-water quality- water sampling and analysis. World Health Organization; 2004 Aug 31
- WHO. How can water-related diseases be prevented during emergencies? Available at: <http://www.who.int/features/qa/31/en/>

1. **% households cooking with clean fuel**

**Responsible Unit:** Environmental Health Office

**Type:** Outcome

**Source (s):** Household Survey

**Type of Reporting:** %

**Interval:** Annually

**References:**

- International Energy Agency (IEA) and the World Bank. 2017. “Progress Towards Sustainable Energy: Global Tracking Framework 2017” (April), World Bank, Washington, DC. Data extracted from <http://gtf.esmap.org/> on 06/20/2017.DC

1. **% children under 5 years of age who are developmentally on track**

**Responsible Unit:** Population and Reproductive Health Office

**Type:** Outcome

**Source (s):** Household Survey

**Type of Reporting:** %

**Interval:** Annually

**References:**

- [UNICEF Early childhood development](https://data.unicef.org/topic/early-childhood-development/development-status/)
- World Health Organization. Primary health care: transforming vision into action: operational framework. World Health Organization; 2018

1. **Malaria incidence**

**Responsible Unit:** Communicable Diseases Office

**Type:** Outcome

**Source (s):** Routine System

**Type of Reporting:** %

**Interval:** Annually

**References:**

- World Health Organization. World malaria report 2015. World Health Organization; 2016 Jan 30

1. **Physical inactivity in adults**

**Responsible Unit:** Non-Communicable Diseases Office

**Type:** Outcome

**Source (s):** Routine System

**Type of Reporting:** Ratio

**Interval:** Annually

**References:**

- WHO's work on physical activity
- Global action plan on physical activity 2018–2030: more active people for a healthier world
- Global recommendations on physical activity for health

1. **Proportion of population subjected to physical, psychological or sexual violence in the previous 12 months**

**Responsible Unit:** Mental Health Office

**Type:** Outcome

**Source (s):** Routine System

**Type of Reporting:** Ratio

**Interval:** Annually

**References:**

- World Health Organization. Global and regional estimates of violence against women: prevalence and health effects of intimate partner violence and non-partner sexual violence. World Health Organization; 2013
- World Health Organization. Responding to intimate partner violence and sexual violence against women: WHO clinical and policy guidelines. World Health Organization; 2013

**Input indicators**

1. **Community/patient participation in facility management meetings**

**Responsible Unit:** Health Network Management

**Type:** Outcome

**Source (s):** Routine System

**Type of Reporting:** %

**Interval:** Annually

**References:**

- PHCPI; Primary Health Care Performance Initiative: Methodology Note, Indicators Library 2015. Available at: <https://improvingphc.org/content/indicator-library>
- Fredriksson M, Tritter JQ. Disentangling patient and public involvement in healthcare decisions: why the difference matters. Sociol Health Illn. 2017;39(1):95-111.
- Alma-Ata. Do. International Conference on Primary Health Care. Alma- Ata; USSR, 6-12

September 1978

**Final Outcome Indicators**

**A- Health Status indicators**

1. **Adolescent mortality rate per 100000**

**Responsible Unit:** Health Network Management

**Type:** Outcome

**Source (s):** Death Registry

**Type of Reporting:** Ratio

**Interval:** Annually

**References:**

- WHO methods for life tables || http://www.who.int/healthinfo/statistics/LT_method.pdf
- World Population Prospects (UN Population Division) || http://esa.un.org/wpp/
- WHO methods and data sources for country-level causes of death, 2000-2015 <http://www.who.int/healthinfo/global_burden_disease/GlobalCOD_method_2000_2015.pdf>

1. **Adult mortality rate 15-60 years per 1000 persons**

**Responsible Unit:** Health Network Management

**Type:** Outcome

**Source (s):** Death Registry

**Type of Reporting:** Ratio

**Interval:** Annually

**References:**

- Methods for estimating adult mortality (UN Population Division, 2002) http://www.un.org/esa/population/publications/adultmort/Complete.pdf
- <http://www.who.int/healthinfo/morttables/en/index.html>
- WHO methods for life tables || http://www.who.int/healthinfo/statistics/LT_method.pdf
- World Population Prospects (UN Population Division)http://esa.un.org/wpp/

1. **Under-five Mortality Rate per 1000 live births**

**Responsible Unit:** Population and Reproductive Health Office

**Type:** Outcome

**Source (s):** Death Registry

**Type of Reporting:** Ratio

**Interval:** Annually

**References:**

- Demographic and Health Surveys (DHS)
- UNICEF, WHO, World Bank, UN DESA/Population Division. Child Mortality Estimates Info database
- World Population Prospects. United Nations, Department of Economic and Social Affairs, Population Division

1. **Neonatal mortality rate 1000 live births**

**Responsible Unit:** Population and Reproductive Health Office

**Type:** Outcome

**Source (s):** Death Registry

**Type of Reporting:** Ratio

**Interval:** Annually

**References:**

- UNICEF, WHO, World Bank, UN DESA/Population Division. Child Mortality Estimates Info database
- Demographic and Health Surveys (DHS)
- World Population Prospects. United Nations, Department of Economic and Social Affairs, Population Division

1. **Infant Mortality Rate 1000 live births**

**Responsible Unit:** Population and Reproductive Health Office

**Type:** Outcome

**Source (s):** Death Registry

**Type of Reporting:** Ratio

**Interval:** Annually

**References:**

- World Population Prospects. United Nations, Department of Economic and Social Affairs, Population Division
- WHO Mortality database
- UNICEF, WHO, World Bank, UN DESA/Population Division. Child Mortality Estimates Info database

1. **Total fertility rate**

**Responsible Unit:** Population and Reproductive Health Office

**Type:** Outcome

**Source (s):** Routine System, Household Survey

**Type of Reporting:** Ratio

**Interval:** Annually

**References:**

- World Population Prospects (UN Population Division)
- OECD (2021), Fertility rates (indicator).

1. **DPT3 Dropout rate**

**Responsible Unit:** Communicable Diseases Office

**Type:** Outcome

**Source (s):** Routine System

**Type of Reporting:** %

**Interval:** Annually

**References:**

- PHCPI; Primary Health Care Performance Initiative: Methodology Note, Indicators Library 2015. Available at: <https://improvingphc.org/content/indicator-library>

1. **TB treatment success**

**Responsible Unit:** Communicable Diseases Office

**Type:** Outcome

**Source (s):** Routine System

**Type of Reporting:** %

**Interval:** Annually

**References:**

- Lozano R, et al. Global and regional mortality from 235 causes of death for 20 age groups in 1990 and 2010: a systematic analysis for the Global Burden of Disease Study 2010. Lancet, 2012, 380:2095–2128
- Broekmans JF et al. European framework for tuberculosis control and elimination in countries with a low incidence: recommendations of the World Health Organization (WHO), International Union Against Tuberculosis and Lung Disease (IUATLD) and Royal Netherlands Tuberculosis Association (KNCV) Working Group. European Respiratory Journal, 2002, 19:765–775

1. **Antenatal care coverage (4+ visits)**

**Responsible Unit:** Population and Reproductive Health Office

**Type:** Outcome

**Source (s):** Routine System, Household Survey

**Type of Reporting:** Ratio

**Interval:** Annually

**References:**

- UNICEF Data: Monitoring the Situation of Children and Women. <http://data.unicef.org/maternal-health/antenatal-care>

1. **People living with HIV receiving anti-retroviral treatment**

**Responsible Unit:** Communicable Diseases Office

**Type:** Outcome

**Source (s):** Routine System

**Type of Reporting:** %

**Interval:** Annually

**References:**

- WHO. HIV/AIDS. Available at: [www.who.int/mediacentre/factsheets/fs360/en/](http://www.who.int/mediacentre/factsheets/fs360/en/)
- WHO. Short summary of ARV guidelines (2013). Available at: <https://www.who.int/hiv/topics/treatment/art/en/>

1. **Prevalence of raised blood pressure (age-standardized estimate)**

**Responsible Unit:** Non-Communicable Diseases Office

**Type:** Outcome

**Source (s):** Household Survey

**Type of Reporting:** Ratio

**Interval:** Annually

**References:**

- World Health Organization. (2007). Prevention of cardiovascular disease: guidelines for assessment and management of total cardiovascular risk. In Prevention of cardiovascular disease: guidelines for assessment and management of total cardiovascular risk. WHO
- World Health Organization. (2007). Prevention of cardiovascular disease: pocket guidelines for assessment and management of cardiovascular risk

1. **Children under 5 with diarrhea receiving ORS**

**Responsible Unit:** Population and Reproductive Health Office

**Type:** Outcome

**Source (s):** Household Survey

**Type of Reporting:** %

**Interval:** Annually

**References:**

- World Health Organization. WHO/UNICEF joint statement. Clinical management of acute diarrhea. Geneva: World Health Organization. 2004
- Gregorio GV, Gonzales ML, Dans LF, Martinez EG. Polymer‐based oral rehydration solution for treating acute watery diarrhea. Cochrane Database of systematic reviews. 2016(12)

**B-Mortality by cause indicators**

1. **Suicide rate**

**Responsible Unit:** Health Network Management

**Type:** Outcome

**Source (s):** Death Registry

**Type of Reporting:** Ratio

**Interval:** Annually

**References:**

- Värnik P. Suicide in the world. International journal of environmental research and public health. 2012 Mar;9(3):760-71.
- Mirhashemi S, Motamedi MH, Mirhashemi AH, Taghipour H, Danial Z. Suicide in Iran. The Lancet. 2016 Jan 2;387(10013):29.

1. **Causes of Death per 100000**

**Responsible Unit:** Health Network Management

**Type:** Outcome

**Source (s):** Death Registry

**Type of Reporting:** Ratio

**Interval:** Annually

**References:**

- World Health Organization. The top 10 causes of death. 2020

1. **Premature NCD mortality % probability**

**Responsible Unit:** Health Network Management

**Type:** Outcome

**Source (s):** Death Registry

**Type of Reporting:** %

**Interval:** Annually

**References:**

- WHO. Global Health Observatory (GHO) dataNCD mortality and morbidity, Available at: <https://www.who.int/gho/ncd/mortality_morbidity/ncd_premature_text/en/>

1. **Life Expectancy at Birth (years)**

**Responsible Unit:** Health Network Management

**Type:** Outcome

**Source (s):** Death Registry

**Type of Reporting:** Ratio

**Interval:** 5-Annual

**References:**

- GHE: Life expectancy and healthy life expectancy
- WHO methods and data sources for life tables,1990-2019

1. **Maternal mortality ratio per 100000 live births**

**Responsible Unit:** Population and Reproductive Health Office

**Type:** Outcome

**Source (s):** Routine System, Sbate-e-Ahval

**Type of Reporting:** Ratio

**Interval:** Annually

**References:**

- Trends in Maternal Mortality: 2000 to 2017 (WHO, UNICEF, UNFPA, World Bank Group and the United Nations Population Division) https://www.who.int/reproductivehealth/publications/maternal-mortality-2017/en

**C-Equality**

1. **Under-five mortality by residence per 1000 live births (Urban/Rural)**

**Responsible Unit:** Health Network Management

**Type:** Outcome

**Source (s):** Death Registry

**Type of Reporting:** Ratio

**Interval:** Annually

**References:**

- UNICEF, WHO, World Bank, UN DESA/Population Division. Child Mortality Estimates Info database
- Estimation methods for child mortality
- World Population Prospects. United Nations, Department of Economic and Social Affairs, Population Division
- WHO Mortality database

1. **Average availability of services for 3 Tracer Communicable Diseases (STI, TB, HIV)**

**Responsible Unit:** Communicable Diseases Office

**Type:** Outcome

**Source (s):** Routine System

**Type of Reporting:** %

**Interval:** Annually

**References:**

- World Health Organization. Service availability and readiness assessment (SARA): an annual monitoring system for service delivery: reference manual. World Health Organization; 2013
- Andriantsimietry SH, Rakotomanga R, Rakotovao JP, Ramiandrison E, Razakariasy ME, Favero R, Gomez P, Dao B, Bazant E. Service availability and readiness assessment of maternal, newborn and child health services at public health facilities in Madagascar. African journal of reproductive health. 2016;20(3):149-58

1. **Perceived access Barriers due to distance**

**Responsible Unit:** Health Network Management

**Type:** Outcome

**Source (s):** Household Survey

**Type of Reporting:** %

**Interval:** Annually

**References:**

- PHCPI; Primary Health Care Performance Initiative: Methodology Note, Indicators Library 2015. Available at: https://improvingphc.org/content/indicator-library
- Al-Taiar A, Clark A, Longenecker JC, Whitty CJ. Physical accessibility and utilization of health services in Yemen. International journal of health geographics. 2010 Dec 1;9(1):38.

1. **Perceived access Barriers due to treatment costs**

**Responsible Unit:** Health Network Management

**Type:** Outcome

**Source (s):** Household Survey

**Type of Reporting:** %

**Interval:** Annually

**References:**

- PHCPI; Primary Health Care Performance Initiative: Methodology Note, Indicators Library 2015. Available at: <https://improvingphc.org/content/indicator-library>
- Kutzin J. Health financing for universal coverage and health system performance: concepts and implications for policy. Bulletin of the World Health Organization. 2013 Jun 17; 91:602-11

1. **Coverage of DPT3 Immunizations (in the first year of life)**

**Responsible Unit:** Communicable Diseases Office

**Type:** Outcome

**Source (s):** Routine System

**Type of Reporting:** %

**Interval:** Annually

**References:**

- Center for Global Development. Making Markets for Vaccines. http://www.who.int/intellectualproperty/news/en/SubmissionBarder1.pdf
- The Childhood Immunization Schedule and Safety: Stakeholder Concerns, Scientific Evidence, and Future Studies (2013)

1. **Care seeking for suspected child pneumonia**

**Responsible Unit:** Population and Reproductive Health Office

**Type:** Outcome

**Source (s):** Household Survey

**Type of Reporting:** %

**Interval:** Annually

**References:**

- Unicef data: pneumonia || https://data.unicef.org/topic/child-health/pneumonia/
- Care Seeking Behaviour for Children with Suspected Pneumonia in Countries in Sub-Saharan Africa with High Pneumonia Mortality (Noordam et al, PLOS Med, 2015). <https://doi.org/10.1371/journal.pone.0117919>

**D-Efficiency**

1. **Adequate waste disposal (Urban/Rural)**

**Responsible Unit:** Environmental Health Office

**Type:** Outcome

**Source (s):** Facility Survey

**Type of Reporting:** %

**Interval:** Annually

**References:**

- Tabrizi JS, Saadati M, Heydari M, Rezapour R, Zamanpour R. Medical waste management improvement in community health centers: an interventional study in Iran. Primary health care research & development. 2019;20
- Graikos A, Voudrias E, Papazachariou A, Iosifidis N, Kalpakidou M. Composition and production rate of medical waste from a small producer in Greece. Waste Management. 2010 Sep 30;30(8):1683-9
- Askarian M, Heidarpoor P, Assadian O. A total quality management approach to healthcare waste management in Namazi Hospital, Iran. Waste management. 2010 30;30(11):2321-6

1. **Provider absence rate**

**Responsible Unit:** Health Network management

**Type:** Outcome

**Source (s):** Facility Survey

**Type of Reporting:** Ratio

**Interval:** Annually

**References:**

- PHCPI: Service Delivery Indicators (SDI). set of health indicators that examine health workers’ effort and ability, as well as the availability of key inputs and resources that contribute to the functioning of a health facility
- Chaudhury, N., J. Hammer, M. Kremer, K. Muralidharan, and F.H. Rogers. (2006). “Missing in Action: Teacher and Health Worker Absence in Developing Countries.” Journal of Economic Perspectives 20(1): 91-116.

**E- Resilience**

1. **Disaster related death rate**

**Responsible Unit:** Health Network management

**Type:** Outcome

**Source (s):** Death Registry

**Type of Reporting:** Ratio

**Interval:** Annually

**References:**

- The International Disaster Database. Brussels: Centre for Research on the Epidemiology of Disasters – CRED
- WHO methods and data sources for country-level causes of death, 2000-2015

**F- Risk Factor/Chronic Disease Prevalence Indicators**

1. **Tobacco use**

**Responsible Unit:** Non-Communicable Diseases Office

**Type:** Outcome

**Source (s):** Health Survey

**Type of Reporting:** %

**Interval:** Annually

**References:**

- U.S. Department of Health and Human Services. [The Health Consequences of Smoking—50 Years of Progress: A Report of the Surgeon General](https://www.cdc.gov/tobacco/data_statistics/sgr/50th-anniversary/index.htm). Atlanta: U.S. Department of Health and Human Services, Centers for Disease Control and Prevention, National Center for Chronic Disease Prevention and Health Promotion, Office on Smoking and Health, 2014 [accessed 2016 Dec 20]

1. **Hypertension prevalence**

**Responsible Unit:** Non-Communicable Diseases Office

**Type:** Outcome

**Source (s):** Health Survey

**Type of Reporting:** Ratio

**Interval:** Annually

**References:**

- World Health Organization. (2007). Prevention of cardiovascular disease: guidelines for assessment and management of total cardiovascular risk. In Prevention of cardiovascular disease: guidelines for assessment and management of total cardiovascular risk. WHO.
- World Health Organization. (2007). Prevention of cardiovascular disease: pocket guidelines for assessment and management of cardiovascular risk

1. **Diabetes Mellitus Prevalence**

**Responsible Unit:** Non-Communicable Diseases Office

**Type:** Outcome

**Source (s):** Health Survey

**Type of Reporting:** Ratio

**Interval:** Annually

**References:**

- Marija Vrca-Botica, Ines Zelić. Quality Indicators for Diabetes Care in Primary Care: as described in The Official Journal of Primary Care Diabetes Europe: Primary Care Diabetes vol. 1, issue 1, 2007
- Canadian Diabetes Association Clinical Practice Guidelines Expert Committee. Canadian Diabetes Association 2003 Clinical Practice Guidelines for the Prevention and Management of Diabetes in Canada. Can J Diabetes. 2003;27(Suppl 2): S1–151. [Ref list]
- Sathira-Angkura T1, Kongsin S, Intaraprasong B, Pattaraarchachai J, Jiamton S. Factors associated with the effectiveness of diabetes care at primary care settings. J Med Assoc Thai. 2011 Dec;94(12):1513-20. Available from: http://www.ncbi.nlm.nih.gov/pubmed/22295741

1. **Obesity prevalence**

**Responsible Unit:** Non-Communicable Diseases Office

**Type:** Outcome

**Source (s):** Health Survey

**Type of Reporting:** Ratio

**Interval:** Annually

**References:**

- World Health Organization. Controlling the global obesity epidemic. Available at: https://www.who.int/nutrition/topics/obesity/en/
- World Health Organization. Diet, nutrition, and the prevention of chronic diseases: report of a joint WHO/FAO expert consultation. World Health Organization; 2003 Apr 22

1. **Average availability of diagnosis and management of 3 tracer NCDs (diabetes, CRD, CVD)**

**Responsible Unit:** Non-Communicable Diseases Office

**Type:** Outcome

**Source (s):** Routine System

**Type of Reporting:** %

**Interval:** Annually

**References:**

- World Health Organization. Service availability and readiness assessment (SARA): an annual monitoring system for service delivery: reference manual. World Health Organization; 2013
- Ghimire U, Shrestha N, Adhikari B, Meheta S, Pokharel Y, Mishra SR. Health system’s readiness to provide cardiovascular, diabetes and chronic respiratory disease related services in Nepal: analysis using 2015 health facility survey

1. **Homicide**

**Responsible Unit:** Health Network management

**Type:** Outcome

**Source (s):** Death Registry

**Type of Reporting:** Ratio

**Interval:** Annually

**References:**

- Method for data collection and validation of data (Violence status report) <http://who.int/violence_injury_prevention/violence/status_report/2014/report/Method_for_data_collection.pdf?ua=1>
- Global status reporting on preventing violence against children 2020 || <https://www.who.int/teams/social-determinants-of-health/violence-prevention/global-status-report-on-violence-against-children-2020>
