# Supplementary material for: Developing Iranian sub-national primary health care measurement framework: a mixed-method study
Source: Arch Public Health. 2023 Jun 1;81:98. doi: 10.1186/s13690-023-01108-0 (PMC10233173; doi:10.1186/s13690-023-01108-0)
Supplement: Supplementary file 2 — Supplementary Material 2 [file 13690_2023_1108_MOESM2_ESM.docx]

System/Structure

1. *Governance Indicators*
   1. *Political commitment and leadership*
2. *Percentage of the Provincial High Council for Health and Food Safety Decisions that have been implemented*
3. *Complaint response rate* *during the first 72 hours*
   1. *Governance and policy frameworks*
4. *Percentage of health facilities with an annual operational plan.*
   1. *Engagement with communities and other multisectoral stakeholders*
5. *Existence of subnational and local strategies for community participation*
6. *Percentage of households with a health ambassador*
   1. *Engagement with private sector provider*
7. *Percentage of health facilities that are managed privately.*
8. *Adjustment to population health needs*
   1. *Monitoring and evaluation*
9. *Priority setting is informed by data & evidence*
10. *Existence of an M&E framework for national health plan meeting criteria*
    1. *PHC oriented research*
11. *Number of applied research projects implemented in the district health network.*
12. *Percentage of public research funding for primary care research*
13. *Finance Indicators -*
    1. *Funding and allocation of financial resources*
14. *Other sources of PHC expenditure (charity) as % of total PHC expenditure*
15. *PHC expenditure as % of total Sub-national health expenditure*
16. *Mental health expenditure as % of total Sub-national PHC expenditure*
17. *Medicine/drugs expenditure as % of total Sub-national PHC expenditure*
    1. *Purchasing and payment systems*
18. *Payment period for HWs working extra hours in the healthcare facility*

Inputs

1. *Physical Infrastructures*
2. *Percentage of PHC facilities with adequate WASH*
3. *Room with auditory and visual privacy for patient consultations*
4. *Facility has access to a computer with email/internet access*
5. *Standard precautions for infection prevention*
6. *Health Workforce*
7. *Percentage of Health workforce in primary care (by occupation)*
8. *Percentage of Primary care workforce specialized in family practice (by occupation)*
9. *Proportion of HWF in PHC have received minimum continuous professional education according to national requirements in the last year*
10. *Vacancy rate in PHC (all levels)*
11. *Density of PHC by occupation (N/10,000 population)*
12. *Medicines and other health products*
13. *PHC EML list correlated to the package of services delivered in PHC*
14. *Proportion of facilities in which essential medicines are available (no stock-outs in X time frame)*
15. *Health Information System*
    1. *Information systems*
16. *Percentage of births registered*
17. *Percentage of deaths registered*
18. *Explicit adoption of a set of PHC indicators for Monitoring and Evaluation*
19. *Inclusion of section on PHC performance in annual health sector reporting*
20. *Percentage of patients who get registered by PHC facilities*
21. *Presence of a comprehensive individual patient/family record*
    1. *Surveillance*
22. *Existence of an effective surveillance system*
23. *Digital technologies for health*
24. *Is there a functioning eHIS in the province/city?*

Process Indicators

1. *Model of care*
   1. *Selection and planning of services*
2. *Annual outpatient department utilization rates per capita*
   1. *Service design*
3. *Percentage of PHC cases referred to secondary care*
   1. *Organization and facility management*
4. *Implementation of the managers' capacity building program, regularly*
   1. *Community linkages and engagement*
5. *Community/patient participation in facility management meetings*
6. *Services for self-care and health literacy in primary care*
7. *Systems for improving quality of care*
8. *Proportion of PHC facilities with up-to-date performance reports in the last 6 months to 1 year*
9. *Percentage of PHC facilities with systems to support quality improvement*
10. *Resilient health facilities and services*
11. *Percentage of facilities meeting criteria for resilient health facilities and services*

Output indicators

1. *Access and availability*
   1. *Accessibility, affordability, acceptability*
2. *Perceived access Barriers due to distance*
3. *Perceived access Barriers due to treatment costs*
   1. *Service availability and readiness*
4. *Provider absence rate*
5. *Percentage of PHC facilities that can provide mental health services*
   1. *Utilization of services*
6. *Percentage of catchment population who received at least one basic visit*
7. *Quality care*
   1. *People-centeredness*
8. *Customer Satisfaction Rate*
9. *Health Worker Satisfaction Rate*
   1. *Effectiveness*
10. *Percentage of registered hypertension patients with BP <140/90 at the last 2 follow-up visits*
11. *Percentage of registered diabetic patients with fasting blood sugar controlled at last 2 follow-up visits/A1C <7%*
12. *Percentage of registered NCD patients with 10 years of cardiovascular risk recorded in the past 1 year*
13. *Children under 5 who are stunted, wasted, overweight, obese*
14. *Percentage of under 5 children that had weight and height measured in the past 1 year*
15. *Percentage of exclusive Breastfeeding 0-5 months*
    1. *Safety*
16. *Number of adverse events reported (immunization)*
17. *Number of adverse events reported (medication)*
18. *Percentage of PHC prescriptions that include antibiotics*
19. *Percentage of PHC prescriptions that include injectable medicines*
20. *Adequate waste disposal*
21. *Percentage of HWs trained on occupational health safety and risk management in the healthcare facility*
22. *Percentage compliance with Hand Hygiene guidelines*
23. *Percentage of trained HWs on Infection Prevention Control*
24. *Percentage of health facilities that have a fire safety and building evacuation program.*
25. *Percentage of HW immunized for Hepatitis B (completed the 3 doses)*
    1. *Efficiency*
26. *Provider case load*
    1. *Timely access*
27. *Average waiting time (min) at PHC facilities*
28. *Percentage of appropriate (upward) referrals during the last 6 months (by specific conditions) with appropriate feedback*

Outcome indicators

1. *Universal health coverage*
   1. *Service coverage*
2. *Percentage of substance users including tobacco users in receipt of brief intervention*
3. *Vaccination of Measles2 and DTP3 (infant-under 23 months age)*
4. *Percentage of women who delivered and received at least once postnatal care within the first 40 days*
5. *Cervical cancer screening rates among women 30-59 years old*
6. *Percentage of Households with adequate WASH*
   1. *Financial protection*
7. *Proportion of population with large/impoverishing household expenditure on health as share of total household expenditure of income*
8. *Health security*
9. *Routine/emergency vaccine (Covid - 19) coverage*
10. *Percentage of Children under 5 years age who are developmentally on track*
11. *Proportion of population subjected to physical, psychological, or sexual violence in the previous 12 months*

Impact

1. *Health Status indicators*
   1. *Good Health and Well-being*
2. *Under-five Mortality Rate per 1000 live births*
3. *Neonatal mortality rate 1000 live births*
4. *Infant Mortality Rate 1000 live births*
5. *Total fertility rate*
6. *Premature NCD mortality % probability*
7. *Maternal mortality ratio per 100000 live births*
8. *Tobacco use*
   1. *Health-related SDGs*
9. *Adult mortality probability 15-60 years per 1000 persons*
10. *Suicide rate*
11. *Causes of Death per 100000*
12. *Disaster related death rate*
13. *Life Expectancy at Birth (years)*
14. *Hypertension prevalence*
15. *Diabetes Mellitus Prevalence*
16. *Obesity prevalence*
17. *TB treatment success*
18. *Antenatal care coverage (4+ visits)*
19. *People living with HIV receiving antiretroviral treatment*
20. *Physical inactivity in adults*
21. *Responsiveness*
22. *Patients’ perceptions of PHC system responsiveness*
23. *Equity*
24. *Average availability of diagnosis and management of 3 tracer NCDs (diabetes, CRD, CVD)*
25. *Under-five mortality by residence per 1000 live births (Urban/Rural)*
26. *Average availability of services for 3 Tracer Communicable Diseases (STI, TB, HIV)*
